# Application of enhanced electronegative multimodal chromatography as the primary capture step for immunoglobulin G purification

**DOI:** 10.1186/s13568-018-0622-3

**Published:** 2018-06-01

**Authors:** Yanli Wang, Quan Chen, Mo Xian, Rui Nian, Fei Xu

**Affiliations:** 1 College of Life Sciences, Jilin University, Changchun, China; 2 CAS Key Laboratory of Biobased Materials, Qingdao Institute of Bioenergy and Bioprocess Technology, Chinese Academy of Sciences, Qingdao, China

**Keywords:** Monoclonal antibody, Electronegative multimodal, Non-affinity purification, Cost-savings

## Abstract

In recent studies, electronegative multimodal chromatography with Eshmuno HCX was demonstrated to be a highly promising recovery step for direct immunoglobulin G (IgG) capture from undiluted cell culture fluid. In this study, the binding properties of HCX to IgG at different pH/salt combinations were systematically studied, and its purification performance was significantly enhanced by lowering the washing pH and conductivity after high capacity binding of IgG under its optimal conditions. A single polishing step gave an end-product with non-histone host cell protein (nh-HCP) below 1 ppm, DNA less than 1 ppb, which aggregates less than 0.5% and an overall IgG recovery of 86.2%. The whole non-affinity chromatography based two-column-step process supports direct feed loading without buffer adjustment, thus extraordinarily boosting the overall productivity and cost-savings.

## Introduction

In biopharmaceuticals, the purification of recombinant immunoglobulin G monoclonal antibodies (IgG mAbs) produced in Chinese hamster ovary (CHO) cells are usually achieved by 3–4 successive chromatographic steps (Girard et al. 2015; Shukla and Thömmes 2010). Affinity chromatography of protein A is extensively used as the primary capture and regarded as the industrial standard (Ghose et al. 2005; Tarrant et al. 2012). Nevertheless, protein A chromatography has its inherent disadvantages, such as relatively low binding capacity, high material/operational cost and ligand leachability, which add another impurity into the process (Shukla et al. 2007; Tao et al. 2014).

Multimodal chromatography has recently emerged as a useful tool for antibody purification. Electronegative multimodal chromatography was even considered as a potential alternative to protein A due to its lower cost and higher NaOH resistance (Urmann et al. 2010; Kaleas et al. 2014). In our recent study, we demonstrated that advance chromatin extraction could significantly improve the dynamic binding capacity (DBC) and IgG recovery of an electronegative multimodal chromatography, Eshmuno HCX. Compared with loading cell culture supernatant (CCS) without chromatin extraction, the DBC of HCX was boosted from 29 to 94 mg/mL, and IgG recovery was also increased from around 80% to over 95% (Gagnon et al. 2014a).

In this study, full DBC profiles of HCX at various pH/salt combinations were systematically characterized. The purification performance of HCX as the primary capture step was significantly enhanced by optimizing the column wash step. Void-exclusion anion exchange chromatography (VEAX) was further integrated with HCX to form a seamless and efficient two-chromatography-step purification process for IgG production.

## Materials and methods

### Reagents and equipment

All chemicals were obtained from Sigma-Aldrich (St. Louis, MO). WorkBeads™ 40 TREN^high^ was purchased from BioWorks (Uppsala, Sweden). Eshmuno^®^ HCX was purchased from Merck Millipore (Merck KGaA, Darmstadt, Germany). UNOsphere™ Q was purchased from Bio-Rad Laboratories (Hercules, CA). Toyopearl AF-rProtein A-650 was purchased from Tosoh Bioscience (Tokyo, Japan). Capto™ adhere was purchased from GE Healthcare (Uppsala, Sweden). Chromatography media were packed in XK or Tricorn™ series columns (GE Healthcare). Chromatography experiments were conducted on an ÄKTA™ Explorer 100 or Avant 25 (GE Healthcare).

### Experimental methods

A biosimilar IgG mAb immunospecific for human epidermal growth factor receptor 2 was produced by CHO cell using a tricistronic vector developed by Ho et al. (2012). Antibody was produced as described in (Gagnon et al. 2014a, b; 2015).

*“Traditional harvest clarification”* was performed by centrifugation at 4000×*g* for 20 min at room temperature, followed by filtration through a 0.22 μm membrane (Nalgene^®^ Rapid-Flow Filters, Thermo Scientific, Waltham, MA). Clarified harvest named as centrifuged/microfiltered CCS (c/m CCS) was stored at 2–8 °C for short-term usage or − 20 °C for long-term storage. Cell culture was alternatively clarified by a more efficient *“advance chromatin extraction”* method developed recently by the research team led by Pete Gagnon (Gagnon et al. 2014a, b, 2015), which was based on the synergistic effect of caprylic acid and allantoin, and harvest clarified by this method was named as chromatin-extracted CCS (c-e CCS).

IgG used for DBC study was highly purified to minimize interference with analytical methods. Protein A affinity chromatography was performed with 20 mL of Toyopearl medium, and eluted IgG was polished by Capto adhere chromatography according to Gagnon et al. (2014a, b, 2015). IgG purified by this process contained < 1 ppm nh-HCP, < 1 ppb DNA and ≤ 0.05% aggregates.

DBCs (mg/mL, at 5% breakthrough) of HCX at different pH/salt combinations were determined by using 4 mL Tricorn 5/10 columns at a linear flow rate of 150 cm/h (2 mL/min, 2 min residence time). The columns were equilibrated with buffers having NaCl from 0 to 200 mM and pH from 6.0 to 4.0, and then stayed off line. The UV detector was zeroed. Highly purified IgG with the same pH and conductivity as the equilibration buffer was pumped into the system until the UV signal at the entrance of the UV monitor matched that in the feed. This UV value was seen to represent 100% breakthrough. The column was then put in-line and monitored until UV signal indicated 5% breakthrough.

IgG capture step was directly performed on HCX chromatography (20 mL medium packed in XK 16/20, 10 cm bed height, at a linear flow rate of 300 cm/h, volumetric flow rate of 10 mL/min). 1500 mL of c-e CCS was loaded to the column pre-equilibrated with equilibration buffer (EQ buffer) (50 mM MES, 100 mM NaCl, pH 6.0), followed by 10 CV of EQ buffer. The column was then washed with 10 CV of 50 mM MES, pH 6.0 or 50 mM acetic acid, pH 5.0 or 50 mM acetic acid, pH 4.0. The column was further washed with 10 CV of EQ buffer, and IgG was eluted with a 5 CV liner gradient to 50 mM Tris, 2.0 M NaCl, pH 8.0 and collected from the point where UV absorbance at 280 nm reached 20 mAU to the point where it descended below that value. The column was sanitized with 5 CV of 1.0 M NaOH. Before storage in 20% ethanol, the column was thoroughly washed with EQ buffer. IgG polishing step was conducted on VEAX mode as described fully in our previous publication (Nian et al. 2013).

### Analytical methods

IgG purity, including nh-HCP, DNA and histone, was documented according to the methods described fully in Gagnon et al. (2014a, 2014b, 2015).

Aggregate content and IgG concentration were measured by analytical size exclusion chromatography (SEC) with a G3000SWxl column (Tosoh Bioscience) on a Dionex Ultimate™ 3000 HPLC system (Thermo Scientific). Residual caprylic acid and allantoin were determined by reversed phase-HPLC (RP-HPLC) (Gagnon et al. 2014a, b, 2015).

Reduced SDS-PAGE was performed on 4–15% Criterion™ TGX Stain-Free™ Gel (Bio-Rad) and stained with a SilverQuest™ Silver Staining Kit from Invitrogen (Carlsbad, CA). Turbidity expressed in nephelometric turbidity units (NTU) was measured with an Orion Q4500 Handheld Turbidity Meter (Thermo Scientific).

## Results

### DBC profiles of HCX at various pH/salt combinations

**Fig. 1:**
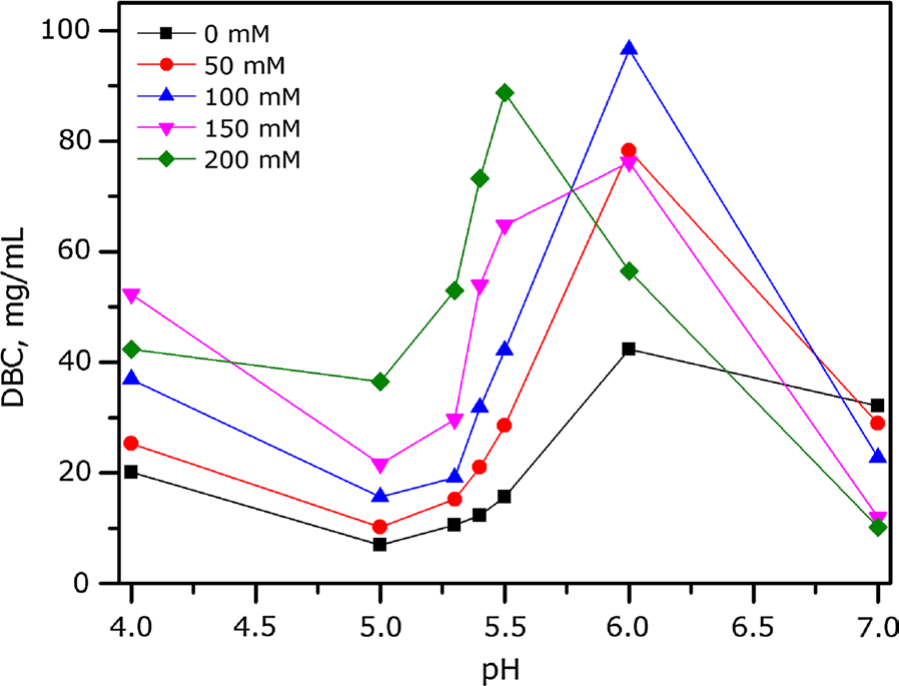
DBC of HCX in response to different pH/salt combinations

Eshmuno HCX medium is described as a multimodal cation exchanger. It is negatively charged due to the sulfo groups (strong ionic) and carboxyl groups (weak ionic). It contains phenyl groups that act in hydrophobic interactions. It also contains hydroxyl and amine groups, exhibiting hydrogen binding properties. These functional groups work together and contribute to the unique DBC profiles of HCX as a function of pH/salt (Fig. 1). The protein adsorption which depends on pH, conductivity and flow rate of the buffer, is regarded as an essential condition to improve the low binding capacities with protein. Unlike traditional strong cation exchangers, which show the strongest binding at low pH and low conductivity (Urmann et al. 2010), HCX demonstrated the highest binding capacity for IgG at pH 6.0 in the presence of 100 mM NaCl. 95 mg/mL DBC was achieved under this optimal condition, which is significantly higher than protein A resins with ≤ 40 mg/mL DBC commercially available on the market (Nian et al. 2016; Urmann et al. 2010) and equivalent to most cation exchangers (Nian and Gagnon 2016).

pH played an essential role in HCX binding capacity to IgG, which should be modulated by the weak ion exchange matrices. The pH had an influence on the ligand density of protein on adsorbent matrixs. At optimal pH value, protein massively utilized bonding site reducing electronic repulsion between different molecules or unsaturation, which made HCX more effective and more flexible to achieve the highest binding capacity of IgG. Both increase and decrease of optimal pH dramatically reduced the DBC of HCX. Notably, at all NaCl concentrations tested, the lowest binding capacity happened at pH 5.0, and it recovered slightly when pH was lower than 5.0. It was also found that, under high-salt conditions, the relative position of the aromatic group was critical to improve the breakthrough capacity, and an amide group on the α-carbon was essential for capturing proteins (Johansson et al. 2003).

### Reducing contaminants binding profiles of HCX

**Fig. 2:**
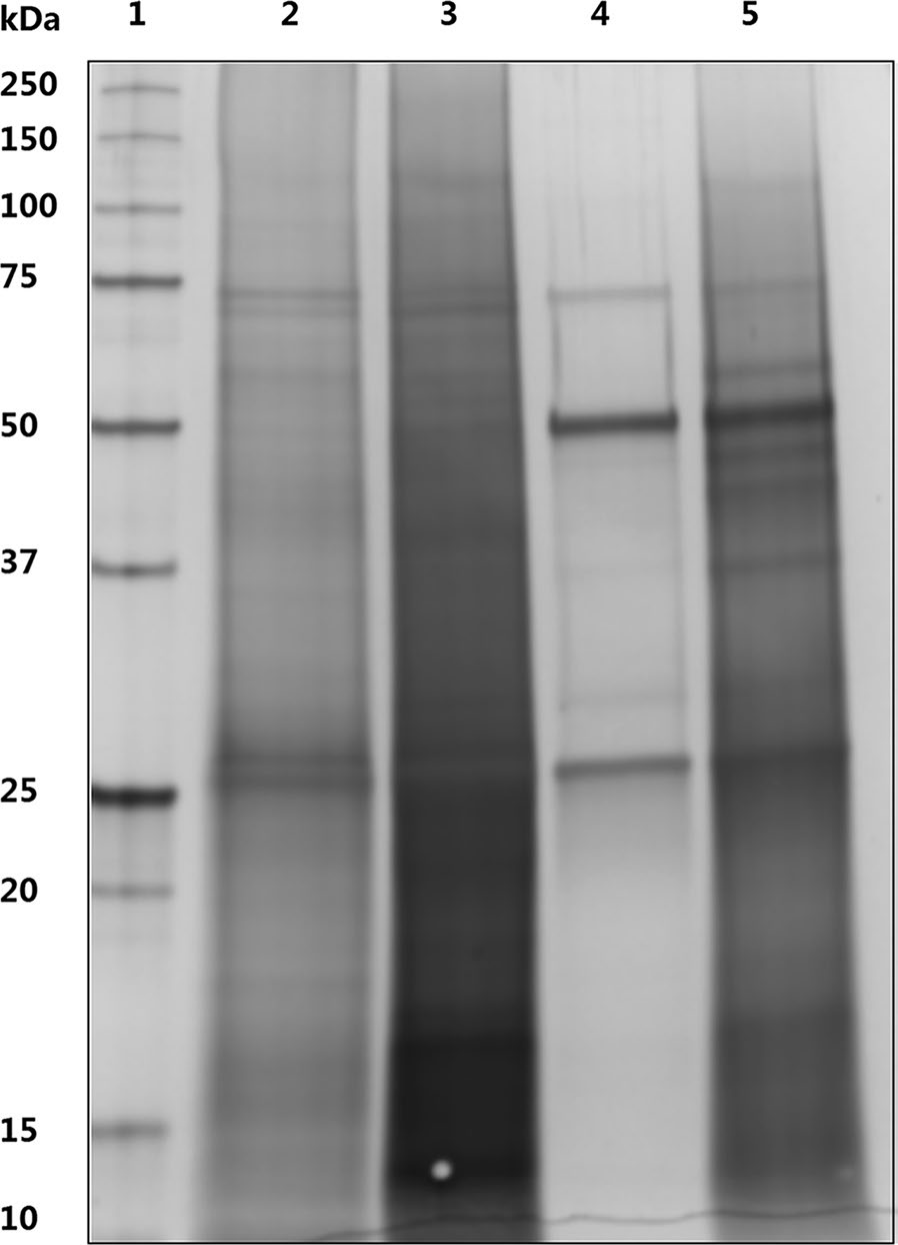
SDS-PAGE in comparison of the NaOH cleaning peak from HCX and protein A chromatography. Lane 1. Molecular weight marker. Lane 2. Supernatant fraction of neutralized 1.0 M NaOH cleaning peak from HCX. Lane 3. Precipitate fraction of neutralized 1.0 M NaOH cleaning peak from HCX. Lane 4. Supernatant fraction of neutralized 0.1 M NaOH cleaning peak from protein A. Lane 5. Precipitate fraction of neutralized 0.1 M NaOH cleaning peak from protein A

It is DNA, histone proteins and nh-HCP that constituted main contaminants in capturing of IgG from CHO. During the whole capture step, IgG was considerably accessible to other dispersive contaminants through elution step even washing step no doubt giving rise to decrease the recovery of IgG. c/m CCS without advance chromatin extraction, which contained all of the impurities mentioned above, was loaded onto HCX and protein A resins separately, and NaOH cleaning fractions of these two chromatographies were compared by SDS-PAGE. As shown in Fig. 2, significant amount of intact IgG was found in protein A cleaning fraction (Lane 4 and Lane 5), which was supposed to be mediated by nonspecific interactions of chromatin with IgG and protein A (Gagnon et al. 2014b, 2015). Contaminant molecules occupied limited bead pools to constrain and hinder IgG bonding with resins. While there was mainly free IgG light chain (LC) and histone components for HCX, compared with protein A, HCX can preferably remove contaminants out of CCS, which found a more high-efficiency methods to capture proteins.

### Optimization of HCX washing conditions to elevate the purity of IgG

**Fig. 3:**
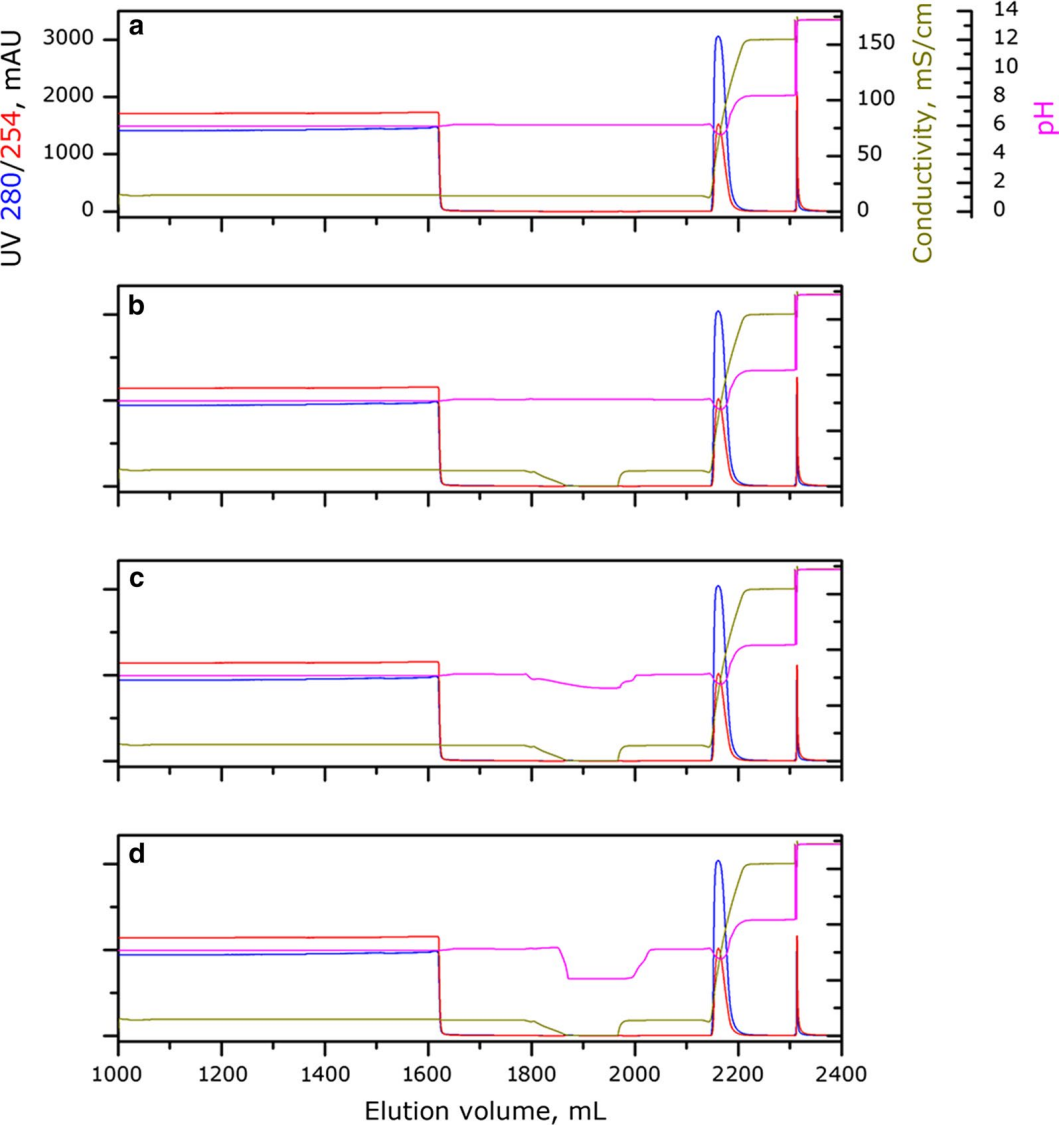
Chromatographic profiles of HCX washed with buffers of different pH and salt concentrations. **a** 200 mM NaCl pH 6. **b** 0 mM NaCl pH 6. **c** 0 mM NaCl pH 5. **d** 0 mM NaCl pH 4

In our previous study, advance chromatin extraction enabled HCX to achieve 94 mg/mL DBC and recover 95% IgG in a sharp peak (Gagnon et al. 2014a, b). We analyzed the influence parameters of elution step above, but not mentioned another crucial step, washing step. In this study, we further optimized the washing steps with buffers at different pH. Interestingly, once IgG was bound onto HCX resin under optimal conditions of pH 6.0 and 100 mM NaCl, lowering pH and salt concentration would not detach bound IgG from the resin, and IgG recovery was around 95% under all testing conditions (Fig. 3a–d). However, nh-HCP was respectively reduced to 1950, 660, 296 and 453 ppm, which proved that the optimized washing strategy could significantly enhance the purification performance of HCX. Contaminate clearance is a significant problem that can’t be ignored through IgG capture. Moreover, the weaker interaction between mAbs and HCX contributed to sufficiently elute proteins avoiding recombination and obstraction. Considering the unique binding properties of HCX, other reported methods to maximize HCP clearance (Ishihara and Hosono 2015; Shukla and Hinckley 2008) may also be worthy to be investigated with HCX in order to achieve more contaminant removal in a single purification step.

### Integrated purification process with two-column-step containing capture and polishing steps

**Fig. 4:**
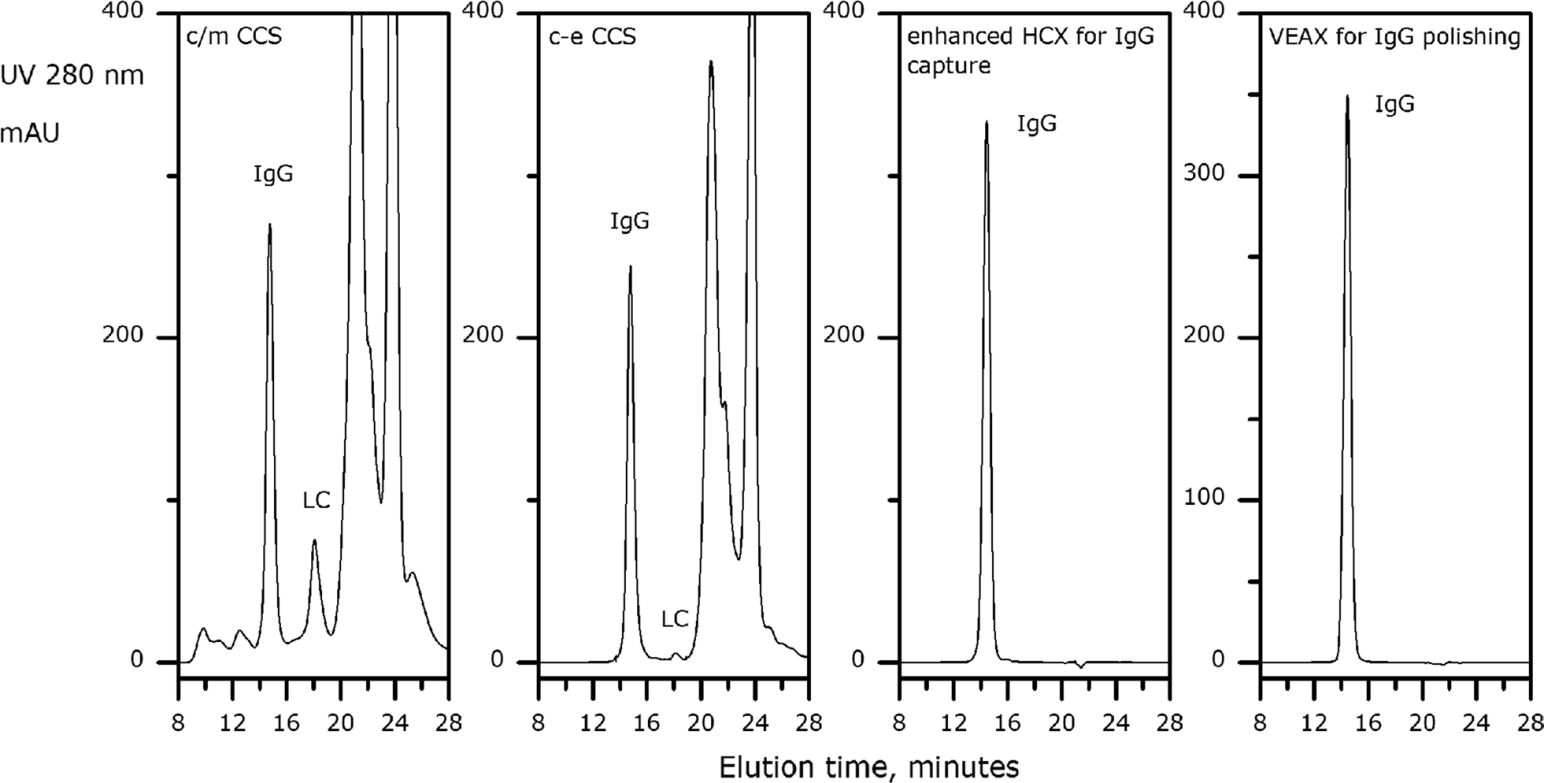
SEC chromatograms for each step of the proposed two-column-step IgG purification platform

VEAX stands for a new mode of anion-exchange chromatography and has been successfully applied as a polishing step for IgG purification with precipitation as the primary capture (Chen et al. 2016). Table 1 summarizes a complete process for IgG purification beginning with advance chromatin extraction, continuing to enhance HCX capture, then a single polishing step with VEAX for further contaminant clearance. Figure 4 illustrates analytical SEC profiles for each process step which included c/m CCS, c-e CCS, enhanced HCX for IgG capture and VEAX for polishing. From the data, we can conclude that many impurities sharp peaks beside IgG peak when using c/m CCS or c-e CCS methods to capture protein. The recovery and purity of IgG can’t have a good performance. While for enhanced HCX for IgG capture and VEAX for polishing methods, they both had a single sharp peak belonged to IgG and the latter one had a higher recovery efficiency of IgG.

**Table 1:** Summary of two-column-step IgG purification process

	CCS	c-e CCS	c-e CCS > enhanced HCX	c-e CCS > enhanced HCX > VEAX
IgG (mg/mL)	1.45	1.12	15.9	10.6
Stepwise IgG recovery (%)	100	91.8	94.6	99.3
DNA (ppm)	10,600	0.05	0.006	0.0004
Histone HCP (ppm)	28,560	< LOD	< LOD	< LOD
nh-HCP (ppm)	224,800	8380	296	0.6
Aggregates (%)	18.65	0.45	0.42	0.40
LC (%)	12.3	1.2	< 0.05	< 0.05
Turbidity (NTU)	25.6	3.11	2.15	1.88
Caprylic acid (μg/mL)	NA	5.67	2.32	0.14
Allantoin (μg/mL)	NA	1119.05	< LOD	< LOD

*NA* not applicable

In the end-product, nh-HCP was reduced to 0.6 ppm, DNA to below 1 ppb and aggregated to less than 0.5%. Residual caprylic acid and allantoin were 0.14 μg/mL and below the limit of detection (LOD, 0.05 μg/mL) respectively. IgG monomer was increased to 99.99% (Fig. 4) with an overall recovery of 86.2%.

## Discussion

Many platform technologies have been developed for IgG mAbs purification. Ion-exchange chromatography (IEX) as one of them was extensively used as a purification technology for mAbs in biochemistry (Ahamed et al. 2007). Interactions between protein and charged substances in IEX were influenced by oppositely charged surfaces of porous chromatographic media. The power of such interactions was supposed to be related to conductivity (Harinarayan et al. 2006). An electronegative multimodal chromatography, a significant branch of IEX, was deeply involved in the proceeding of IgG purification throughout the research.

Traditional harvest clarification in upstream process needs to experience a series of complicated operations, such as centrifugation and filtration, consuming a long period of time. A cutting edge method called advance chromatin extraction plays the same clarified role as traditional harvest clarification with more efficient harvest. Antibodies obtained above were directly captured through electronegative multimodal resin, Eshmuno HCX, with different pH/salt combinations to analyze DBCs parameter of HCX. The results presented in this study demonstrated a satisfactory performance of HCX to utilize for IgG purification. It was further systematically characterized for its IgG binding capacity at various pH/salt combinations. Protein DBCs which were demonstrated as a function of pH and conductivity on resin were expected to decrease with increasing conductivity and decreasing protein charge (Harinarayan et al. 2006). Although the resin supports the highest binding capacity at pH 6.0 with 100 mM NaCl, lowering pH and salt concentration would not detach the bound IgG.

The current process supports direct feed loading for both capture and polishing steps without buffer adjustment called VEAX. Both HCX and VEAX resins are able to withstand the exposure to 1.0 M NaOH which leads to lower bioburden, longer cycle life and decreased validation cost compared to other purification platforms based on protein A or the combination of ion exchangers (Ahamed et al. 2007; Kröner et al. 2013) and hydrophobic interaction chromatography (HIC) (Queiroz et al. 2001; Baumann et al. 2015). Therefore the two-column-step process proposed in this study provides a promising alternative for IgG purification at first step. The current study had explored the effect of pH/salt combinations on the DBC of IgG. It was seen to be related to conductivity and resin ligand density. Different pH/salt components could adjust the ligand density with proteins. The optimal condition can make full use of space on the resin to keep the protein molecules on balance without hindering and repelling of protein charge.

In this work, nh-HCP, DNA and histone proteins regarded as primary impurities should be carefully focused to be removed out of purified IgG. Compared with protein A resins, HCX made a better performance in detaching histone from IgG illustrated in SDS-PAGE (Fig. 2). Once IgG mAbs bound with HCX matrixs in the optimal pH no matter how to change the pH values of washing buffer, IgG still combined with resins. While alter washing step not only limited in pH but also contained components and washing methods, all of them could reduce impurities. Enhanced washing steps were thus developed and demonstrated to be effective to elevate HCP removal. Eluted IgG was directly loaded onto VEAX to further remove HCP to below 1 ppm, DNA below 1 ppb and aggregates less than 0.5% with an overall IgG recovery of 86.2%. This new two-column-step purification platform supports direct IgG capture from c-e CCS and polishing with VEAX without buffer adjustment. It overcomes the traditional limitations of protein A chromatography, and can help boost productivity and cost-savings.

Protein A chromatography as a highly robust technology was also significantly deficient in high cost and low productivity (Dutta et al. 2015). Developing technologies for overcoming the drawbacks of existing methods was of great importance to purify IgG mAbs which were produced for commercial products interested in therapeutic treatments for numerous diseases. Ion-exchange chromatography was currently used as a part of mAbs purification process to effectively remove out of contaminants within CCS. In this study, it illustrated that cost-saving ion-exchange chromatography had the potential to replace protein A chromatography as the first step in capturing IgG.

## Abbreviations

IgG: immunoglobulin G
nh-HCP: non-histone host cell protein
IgG mAbs: immunoglobulin G monoclonal antibodies
CHO: Chinese hamster ovary
DBC: dynamic binding capacity
CCS: cell culture supernatant
VEAX: void-exclusion anion exchange chromatography
c/m CCS: centrifuged/microfiltered CCS
c-e CCS: chromatin-extracted CCS
EQ buffer: equilibration buffer
SEC: size exclusion chromatography
RP-HPLC: reversed phase-HPLC
LOD: limit of detection
NTU: nephelometric turbidity units
HIC: hydrophobic interaction chromatography
IEX: ion exchange chromatography

## References

[CR1] Ahamed T, Nfor BK, Verhaert PD, van Dedem GW, van der Wielen LA, Eppink MH, van de Sandt EJ, Ottens M (2007). pH-gradient ion-exchange chromatography: an analytical tool for design and optimization of protein separations. J Chromatogr A.

[CR2] Baumann P, Baumgartner K, Hubbuch J (2015). Influence of binding pH and protein solubility on the dynamic binding capacity in hydrophobic interaction chromatography. J Chromatogr A.

[CR3] Chen Q, Abdul Latiff SM, Toh P, Peng X, Hoi A, Xian M, Zhang H, Nian R, Zhang W, Gagnon P (2016). A simple and efficient purification platform for monoclonal antibody production based on chromatin-directed cell culture clarification integrated with precipitation and void-exclusion anion exchange chromatography. J Biotechnol.

[CR4] Dutta AK, Tran T, Napadensky B, Teella A, Brookhart G, Ropp PA, Zhang AW, Tustian AD, Zydney AL, Shinkazh O (2015). Purification of monoclonal antibodies from clarified cell culture fluid using Protein A capture continuous countercurrent tangential chromatography. J Biotechnol.

[CR5] Gagnon P, Nian R, Tan L, Cheong J, Yeo V, Yang Y, Gan HT (2014). Chromatin-mediated depression of fractionation performance on electronegative multimodal chromatography media, its prevention, and ramifications for purification of immunoglobulin G. J Chromatogr A.

[CR6] Gagnon P, Nian R, Lee J, Tan L, Latiff SM, Lim CL, Chuah C, Bi X, Yang Y, Zhang W, Gan HT (2014). Nonspecific interactions of chromatin with immunoglobulin G and protein A, and their impact on purification performance. J Chromatogr A.

[CR7] Gagnon P, Nian R, Yang Y, Yang Q, Lim CL (2015). Non-immunospecific association of immunoglobulin G with chromatin during elution from protein A inflates host contamination, aggregate content, and antibody loss. J Chromatogr A.

[CR8] Ghose S, Allen M, Hubbard B, Brooks C, Cramer SM (2005). Antibody variable region interactions with Protein A: implications for the development of generic purification processes. Biotechnol Bioeng.

[CR9] Girard V, Hilbold NJ, Ng CK, Pegon L, Chahim W, Rousset F, Monchois V (2015). Large-scale monoclonal antibody purification by continuous chromatography, from process design to scale-up. J Biotechnol.

[CR10] Harinarayan C, Mueller J, Ljunglöf A, Fahrner R, Van Alstine J, van Reis R (2006). An exclusion mechanism in ion exchange chromatography. Biotechnol Bioeng.

[CR11] Ho SC, Bardor M, Feng H, Mariati Tong YW, Song Z, Yap MG, Yang Y (2012). IRES-mediated tricistronic vectors for enhancing generation of high monoclonal antibody expressing CHO cell lines. J Biotechnol.

[CR12] Ishihara T, Hosono M (2015). Improving impurities clearance by amino acids addition to buffer solutions for chromatographic purifications of monoclonal antibodies. J Chromatogr B Analyt Biomed Life Sci.

[CR13] Johansson BL, Belew M, Eriksson S, Glad G, Lind O, Maloisel JL, Norrman N (2003). Preparation and characterization of prototypes for multi-modal separation aimed for capture of positively charged biomolecules at high-salt conditions. J Chromatogr A.

[CR14] Kaleas KA, Tripodi M, Revelli S, Sharma V, Pizarro SA (2014). Evaluation of a multimodal resin for selective capture of CHO-derived monoclonal antibodies directly from harvested cell culture fluid. J Chromatogr B Analyt Technol Biomed Life Sci.

[CR15] Kröner F, Hanke AT, Nfor BK, Pinkse MW, Verhaert PD, Ottens M, Hubbuch J (2013). Analytical characterization of complex, biotechnological feedstocks by pH gradient ion exchange chromatography for purification process development. J Chromatogr A.

[CR16] Nian R, Gagnon P (2016). Advance chromatin extraction enhances performance and productivity of cation exchange chromatography-based capture of immunoglobulin G monoclonal antibodies. J Chromatogr A.

[CR17] Nian R, Chuah C, Lee J, Gan HT, Latiff SM, Lee WY, Vagenende V, Yang YS, Gagnon P (2013). Void exclusion of antibodies by grafted-ligand porous particle anion exchangers. J Chromatogr A.

[CR18] Nian R, Zhang W, Tan L, Lee J, Bi X, Yang Y, Gan HT, Gagnon P (2016). Advance chromatin extraction improves capture performance of protein A affinity chromatography. J Chromatogr A.

[CR19] Queiroz JA, Tomaz CT, Cabral JMS (2001). Hydrophobic interaction chromatography of proteins. J Biotechnol.

[CR20] Shukla AA, Hinckley P (2008). Host cell protein clearance during protein A chromatography: development of an improved column wash step. Biotechnol Prog.

[CR21] Shukla AA, Thömmes J (2010). Recent advances in large-scale production of monoclonal antibodies and related proteins. Trends Biotechnol.

[CR22] Shukla AA, Hubbard B, Tressel T, Guhan S, Low D (2007). Downstream processing of monoclonal antibodies—application of platform approaches. J Chromatogr B.

[CR23] Tao Y, Ibraheem A, Conley L, Cecchini D, Ghose S (2014). Evaluation of high-capacity cation exchange chromatography for direct capture of monoclonal antibodies from high-titer cell culture processes. Biotechnol Bioeng.

[CR24] Tarrant RD, Velez-Suberbie ML, Tait AS, Smales CM, Bracewell DG (2012). Host cell protein adsorption characteristics during protein A chromatography. Biotechnol Prog.

[CR25] Urmann M, Graalfs H, Joehnck M, Jacob LR, Frech C (2010). Cation-exchange chromatography of monoclonal antibodies: characterisation of a novel stationary phase designed for production-scale purification. MAbs.

